# Theory and practice in medical education – expectations and development of skills experienced by students of human medicine compared with students in other disciplines

**DOI:** 10.3205/zma000950

**Published:** 2015-02-11

**Authors:** Silke Piedmont, Bernt-Peter Robra

**Affiliations:** 1 Otto-von-Guericke University Magdeburg, Institute for Social Medicine and Health Economics, Magdeburg, Germany

**Keywords:** medical education, study, scientific orientatio, practical relevance, skills, career expectations, aim of studies, principles of teaching

## Abstract

**Aim: **The aim of this article is to compare students of human medicine (HM) with students specialising in the MINT disciplines (mathematics, computer science, natural sciences and engineering), the humanities and social sciences as well as law and economic sciences with regard to their expectations of their university study and career and the areas of competence where they feel they have been supported by their education. We present in detail issues particularly relevant to prospective physicians, which are discussed with the main focus on the “theoretical and practical orientation of medical education”.

**Methods: **We used the database in the Public Use File of the “11^th^ Student Survey”, a written survey of randomly selected students studying at 25 German tertiary institutions during the 2009/2010 winter term, which was supplied by the Tertiary Education Research working group at the University of Constance. Data on 7536 students was included, of which 488 (6.5%) were prospective physicians.

**Results: **Human medicine students have a clear career aim and want to complete their education quickly. They have a far above-average interest in working with and for people. About one student in two is interested in a career in science or research (53% in each case – close to the average for all subjects). Compared with the other disciplines, HM students are most likely to consider their university education to have practical and research relevance and are most likely to feel prepared for their profession. Yet over half of all students (Ø 53.3%; HM 54.5%) do not consider their education to have fostered their research skills. MINT students in particular are better able to enhance their skills through independent experimentation, while theory and practice are more likely to be communicated academically in the regular teaching of human medicine. Accordingly, the HM students feel less well supported in some areas of competence required for their later work than students in other disciplines, in developing independence, problem-solving ability, critical ability and capacity for teamwork for example.

**Conclusion: **The high expectations held by human medicine students of being prepared for practical work with/on people are met to an above-average degree according to their assessments of the “practical relevance” and “career preparation” offered by their medical education. However the perceived development of skills in theory and practice does not respond sufficiently well to the demands of the complex, responsible profession they aspire to. Medical students should be better supported in developing both practical and academic independence.

## Zusammenfassung

**Zielsetzung:** Der vorliegende Artikel vergleicht Studierende der Humanmedizin (HM) mit Studierenden der Fächergruppen MINT, Geistes- und Sozialwissenschaften sowie Rechts- und Wirtschaftswissenschaften hinsichtlich ihrer Erwartungen an Studium und Beruf und der Kompetenzbereiche, in denen sie sich im Studium gefördert fühlen. Dadurch werden Spezifika der angehenden Humanmediziner/innen herausgearbeitet, die unter dem Fokus „Wissenschafts- und Praxisorientierung des Studiums“ diskutiert werden.

**Methodik: **Datenbasis ist der Public Use File des „11. Studierendensurveys“, eine schriftliche Befragung von zufällig ausgewählten Studierenden an 25 deutschen Hochschulen im Wintersemester 2009/2010, die durch die Arbeitsgruppe Hochschulforschung der Universität Konstanz zur Verfügung gestellt wurde. Angaben von 7536 Studierenden werden einbezogen, darunter 488 (6,5%) angehende Humanmediziner/innen.

**Ergebnisse: **Humanmedizinerinnen und Humanmediziner haben ein klares Berufsziel und möchten zügig studieren. Sie weisen ein weit überdurchschnittliches Interesse an der Arbeit mit und für Menschen auf. Rund jede/r zweite von ihnen ist an einer wissenschaftlichen oder forschenden Tätigkeit interessiert (je 53% - nahe am Durchschnitt aller Fächer). Verglichen mit den anderen Fächergruppen sprechen HM ihrem Studium am häufigsten einen Praxis- sowie Forschungsbezug zu und fühlen sich am häufigsten auch auf ihren Beruf vorbereitet. Jedoch sieht sich über die Hälfte aller Studierenden (Ø 53,3%; HM 54,5%) eher nicht in ihrer Forschungskompetenz gefördert. Vor allem MINT-Studierende können ihre Kompetenzen stärker durch selbständiges Erproben erweitern, während Praxis und Wissenschaft in der regulären Lehre der Humanmedizin eher theoretisch vermittelt werden. Entsprechend fühlen sich die HM in einigen Kompetenzbereichen, die für ihre spätere Arbeit nötig sind, weniger gefördert als Studierende anderer Fächergruppen, beispielsweise in ihrer Selbständigkeit, Problemlösungsfähigkeit, Kritik- und Teamfähigkeit.

**Schlussfolgerung: **Die hohen Erwartungen der Humanmedizin-Studierenden an eine Vorbereitung auf die praktische Tätigkeit mit/am Menschen werden nach ihren Bewertungen des „Praxisbezuges“ und der „Berufsvorbereitung“ im Studium überdurchschnittlich gut erfüllt. Dennoch entspricht die wahrgenommene Kompetenzförderung in Praxis und Wissenschaft nicht ausreichend dem komplexen verantwortungsvollen Beruf, den sie anstreben. Studierende der Medizin sollten in ihrer praktischen und wissenschaftlichen Selbständigkeit stärker gefördert werden.

## Introduction

§ 1 of the regulations for licensing doctors [1] postulates: *“The aim of medical education is a doctor educated in both the practice and theory of medicine*”. The duality of theory and practice in education is not restricted to the study of medicine. 

The practical orientation of medical education refers to the graduates’ activities in potential professional fields (see [2]). Practice-oriented study is not restricted to preparation for a profession however: it promotes independent action (i. e. πραξις) and experimentation by the students. Practical orientation can be identified not only in teaching content but also in teaching methods.

“Scientifically oriented teaching and learning processes” as defined by the German Education Council does not mean, “…that teaching should be geared towards scientific activity or even research.” ([3], p. 33). On the contrary, it should be understood in terms of the following: systematic and theory-based thinking, critical appraisal, assessment of complicated and contradictory information, students’ ability to develop their own questions and their application, students’ ability to produce, present and defend their own findings and the discovery of the limits of scientific knowledge (see [4], p. 3f); [5]). Methods and processes such as “exploratory learning, independent and cooperative working, training in problem solving” are considered by the German Education Council in this context as “equally important as the course content itself” ([3], p. 133).

Scientific and practical orientation can be mutually enriching (see [6], [7], [8], [3]). For example, practical phases of the course, at least when they are effectively designed, set up and supported, can also communicate basic scientific skills [9], [10]. The regulations for licensing doctors attempt in their code to combine the communication of theoretical and clinical knowledge *“during the whole of medical education to the fullest possible extent"* (§ 2 par. 2) in order to prevent the “compartmentalisation of knowledge”, and to avoid a situation where “*”formal education” on the one hand and “know-how” on the other co-exist peacefully without causing mutual stimulation*” ([11], p. 3). A scientific and practical orientation, successfully implemented, enables graduates to reflect on and improve their professional work and its requirements [4], [12], (see [13]).

The expectations that students of human medicine (HM) have of their university education and professional life have already been the subject of research [14], [15], [16]. Only a comparison with students from other disciplines, however, can show which expectations and problems are common to all disciplines and thus tend to be typical of students or of the tertiary education system, and which relate more specifically to human medicine i.e. may constitute a need for action in medical education. In the few publications that compare disciplines on the basis of random samples of this kind, human medicine is generally combined with other health-related courses [17], [7] and/or specific analyses of human medicine [18] undertake a few interdisciplinary comparisons only without the focus on “science and practice”. The aim of this analysis is to find out where human medicine courses should be optimised from the students’ point of view and what suggestions other disciplines offer for this. The students’ expectations of their course and professional life are presented first, followed by the practical, scientific and cross-disciplinary skills that they feel are promoted. The primary source did not classify its questions in this way. In the presentation below, the responses are left in context. For this reason, it is impossible to avoid referring forwards and backwards in the text to some degree.

## Methods

### Data base

The data base used is the “11^th^ Student Survey”, a written survey of 7,590 randomly selected students (response rate 27.8%) conducted at 25 German tertiary institutions in the 2009/10 winter term [19], [20]^1^. Some of the items underwent descriptive analysis in a special analysis for the Medical Faculty Association [18].

Students are assigned to their first-named (major) subject. Departing from publications to date on the 11^th^ Student Survey, all students who have completed a first degree are also considered (n=927), because they may have a particularly detailed picture of their current degree course. However, the data set is cleaned up to eliminate anybody who did not provide usable information on their subject (n=115) or gave human medicine as their first subject but named no final degree consistent with HM or with their second degree (n=3). While the analysis by Kolbert-Ramm/Ramm [18] considered only those human medicine students aiming for the state examination, students are included who are studying human medicine as their first subject and state, in connection with their *next* degree, that

- they aim to take the state examination (n=472) or undertake a doctoral qualification (because re-enrolment as a doctoral student is an option immediately after the state examination; n=14) or
- they want to complete a different next degree, if they name a compatible second or third degree programme (n=2).

Accordingly a total of 7,536 respondents are considered, including 488 HM students from institutions in Bochum, Dresden, Duisburg-Essen, Frankfurt, Freiburg, Hamburg, Leipzig, Magdeburg, Munich and Rostock. Three people failed to give a location. Five people are studying another subject besides human medicine. Of the HM students, 47.0% are in their 1^st^-4^th^ semester, 50.5% in later semesters and 2.4% do not specify the semester.

#### Selection of comparison groups

The responses by the HM students are contrasted with those obtained from students in three subject groups:

- Mathematics, computer science, natural sciences and engineering (MINT, 36.2% of the random sample)
- Humanities and social science (GSW, 37.8%)
- Law and economic sciences (RWW, 19.6%).

For the 13 fields of study broadly categorised in the official statistics [21], see [19], the following mapping was the result of a cluster analysis with the characteristics of the question *“What aspect of a profession is especially important for you personally?"*

- for GSW: languages and arts, psychology, sports science/sports education, social sciences and education, medicine excluding human medicine (i.e. dentistry/veterinary medicine, health sciences/health management/health education, professions allied to medicine, nursing studies), art/fine arts, music
- for RWW: law, industrial engineering, business informatics, economic sciences
- for MINT: mathematics, natural sciences, agricultural sciences, forestry and nutritional sciences, engineering.

#### Sociodemographic characteristics of the sample and population

The sociodemographic characteristics of the participants in the four subject groups are presented in the supplement (see Table 1 (Tab. 1)). It was no surprise that a higher semester is mentioned on average in human medicine because of the comparatively long standard period of study. The highest proportion of women was found in GSW and HM. The percentage distribution of subject groups in the student survey deviates only slightly from the student population in the same year (see Table 2 (Tab. 2), see [21]). In all subject groups, a higher percentage of women responded than would have been expected in relation to the population (HM +9.5% points, Ø +8.8% points).

#### Data analysis

Data were analysed with SPSS (version 21) statistical software. Missing data were excluded casewise. Statistical tests were considered significant at a probability of p≤0.05. If no significance level is given in the comparison of the four subject groups in the results section, the p-value is lower than 0.001. The Kruskal-Wallis test was applied for comparisons of mean values. When Likert scales with scores 0-6 were used; scores 0-2 and 4-6 were generally combined and score 3 left as “indifferent”.

## Results

### Motivation for studying and professional motivation

**Figure 1:**
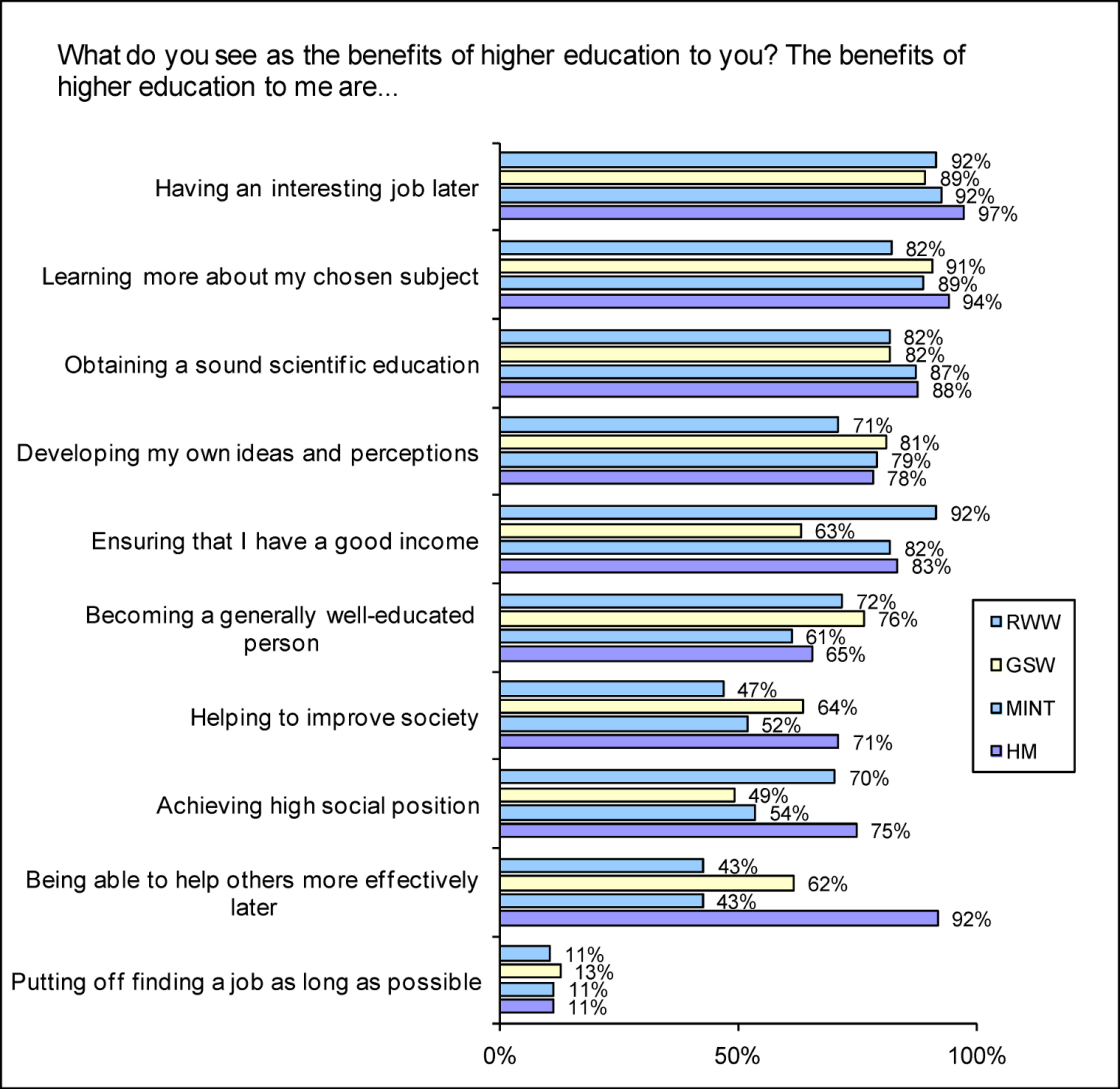
Responses to the question “What do you see as the benefits of higher education to you? The benefits of higher education to me are…” (Proportion of scores 4-6 on a scale going from 0=not beneficial to 6= highly beneficial)

Students from all disciplines are most likely to see the benefit of their own higher education in terms of their ability to find an interesting job later (see Figure 1 (Fig. 1)). Prospective physicians are far more likely than other students to perceive the benefit in the ability to help people (HM: 91.8%; Ø 53.0%) and to help to improve society (HM: 71.1%; Ø 56.6%). HM students were more likely than average to expect a sound scientific education (HM: 87.7%; Ø 84.2%). In contrast, they had lower than average expectations of becoming a person with a good general education (HM: 65.7%; Ø 69.3%). Prospective physicians, followed by RWW, are more likely than other students to regard their education as a means to high social position (HM: 74.8%; Ø: 56.6%).

**Figure 2:**
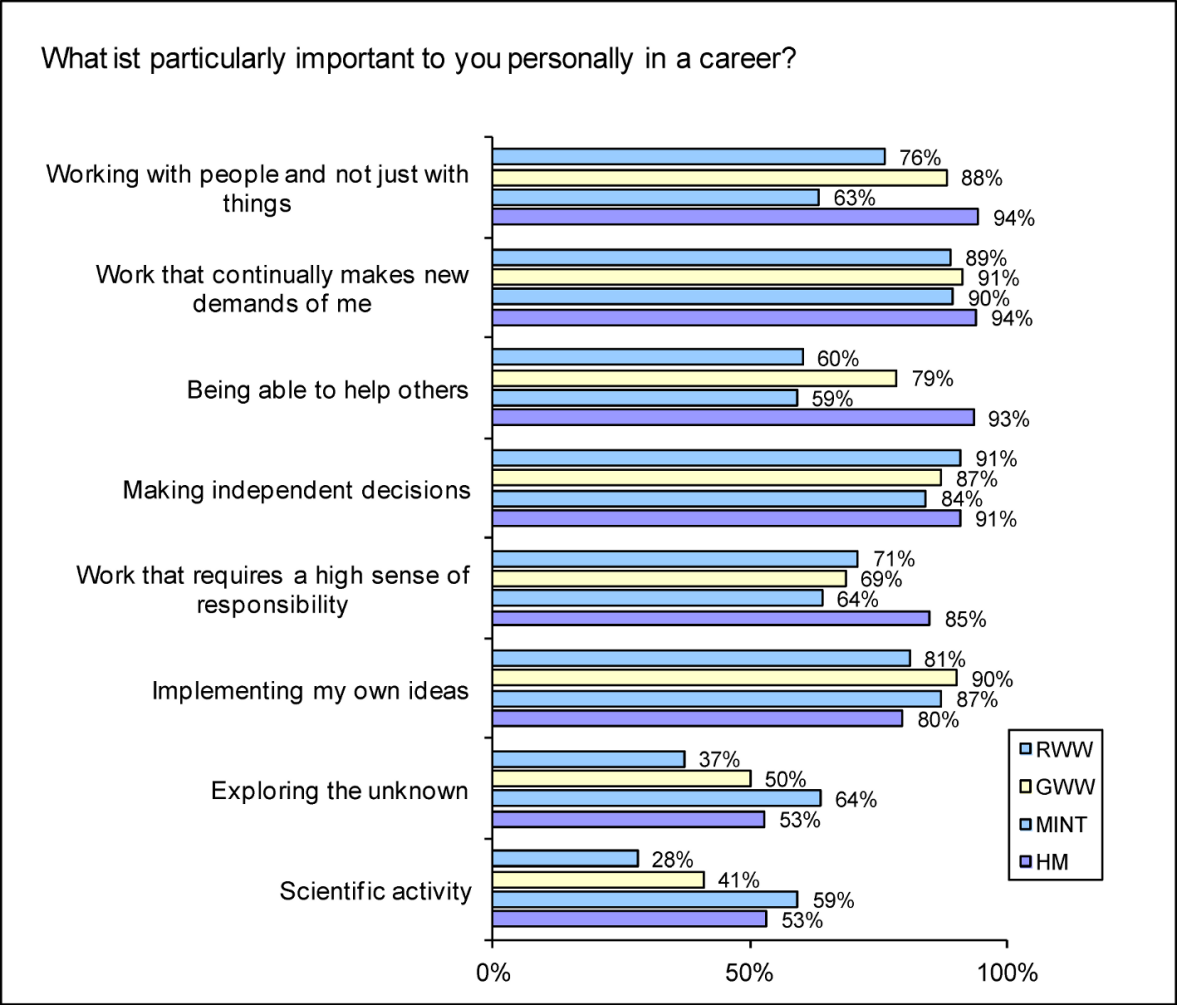
Selected responses to the question “What is particularly important to you personally in a career?” (Proportion of scores 4-6 on a scale going from 0= “totally unimportant” to 6= “very important”)

Only about half of the total student population in the four subject groups is interested in scientific and research activities (scientific Ø 45.8%, research Ø 52.6%: see Figure 2 (Fig. 2)), with the MINT students the most interested. About one in two of the prospective physicians thinks it is important to engage in scientific activities and/or to explore the unknown in professional life.

Developing and implementing their own ideas, an important part of scientifically oriented education, is of below-average significance for human medicine students (79.7%; Ø 86.7%). On the other hand, making independent decisions in their professional life is of above-average significance for them (HM: 90.9%, Ø 87.1%).

**Figure 3:**
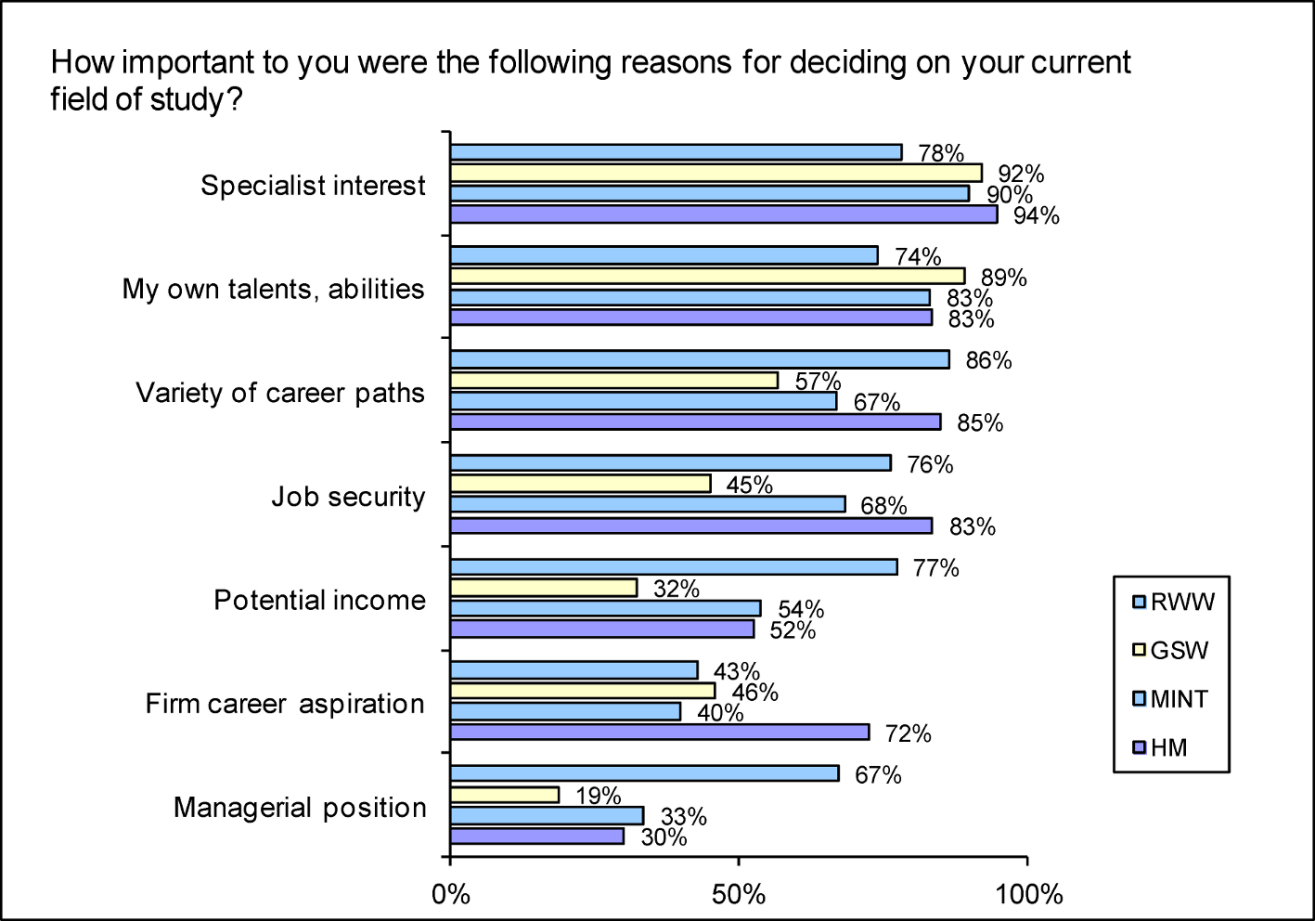
Responses to the question “How important to you were the following reasons for deciding on your current field of study?” (Proportion of scores 4-6 on a scale going from 0= “totally unimportant” to 6= “very important”)

As reasons for choice of university course, students were most likely to name special professional interest as important (Ø 88.6%). For the students of medicine, the range of potential career paths was particularly significant (HM: 84.8%, Ø 68.0%), as was job security (HM: 83.4%; Ø 61.9%). They are substantially more likely than the others to have a firm career aspiration in mind but tend to show average interest in income, promotion and managerial responsibility (see Figure 3 (Fig. 3) and long-version Figure 2 see attachment ) – for law and economic science students, these are substantially more important reasons.

In addition, students were asked about the importance of completing their studies quickly, from which students of human medicine and of RWW were more likely than average to expect advantages for their intellectual/personal development (RWW: 64.4%; HM: 60.4%, Ø 58.9%) and for their career prospects (RWW: 88.7%; HM: 84.3%; Ø: 83.9%; no figure).

#### Promoting practical skills

**Figure 4:**
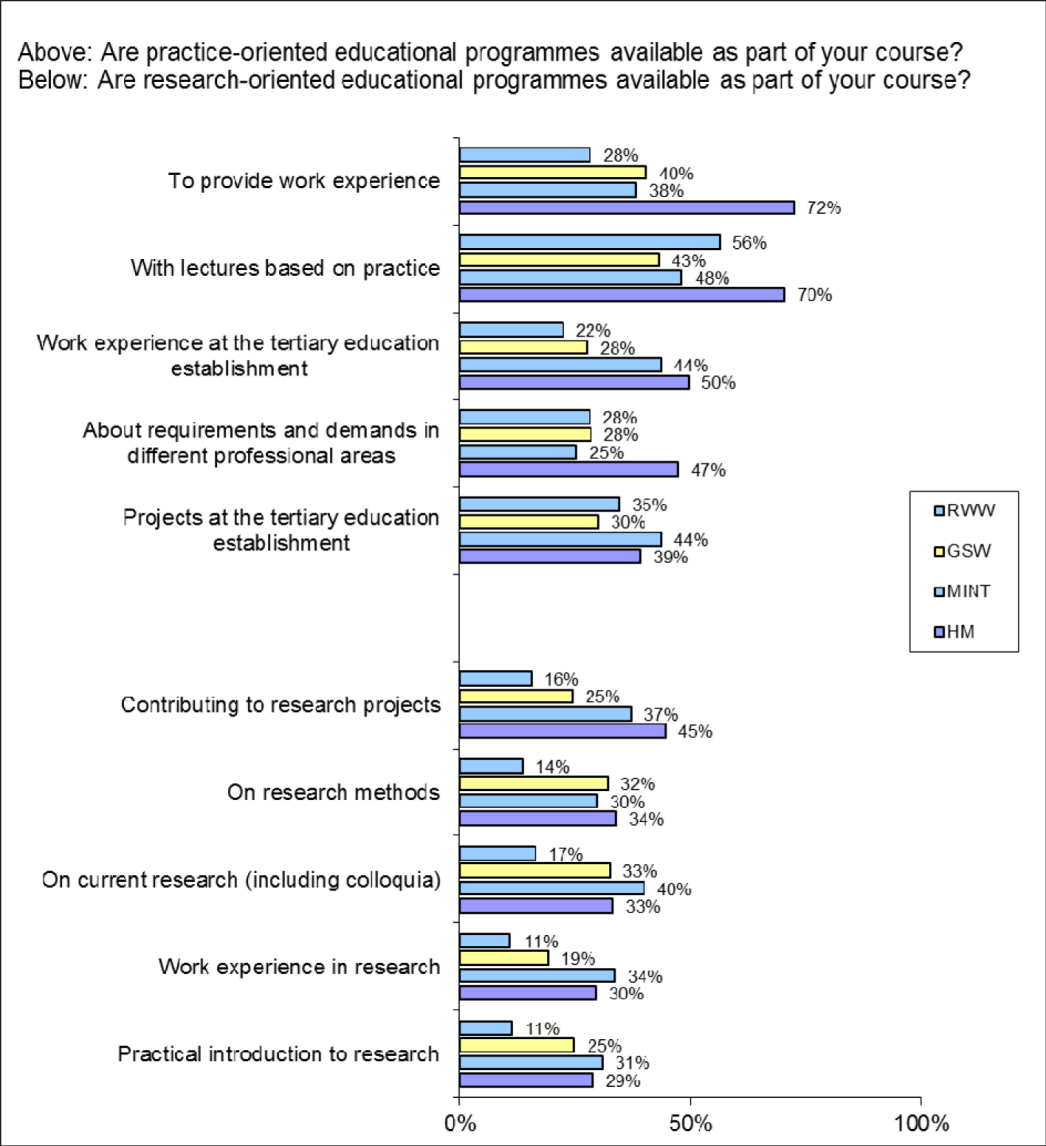
Responses to the questions “Are practice-oriented educational programmes available as part of your course?” (above) and “Are research-oriented educational programmes available as part of your course?” (below) (Proportion of scores 4-6 on a scale from 0-6 where 6=”totally applicable”)

The majority of medical students state that they have already completed a work placement in Germany (85.0%) and outside Germany (22.5%) – the MINT students are the least likely to have done so. Because only half of HM students state that work experience is offered at the tertiary education establishment (see Figure 4 (Fig. 4)), the high scores for completed work experience must be based to a substantial degree on opportunities outside university (nursing, clinical traineeship). *Educational programmes providing work experience* are most likely to be reported in human medicine (HM 72.3%; Ø 39.3%); the same applies to *Lectures based on practice* (HM 70.3%; Ø 49.3%;). Practice-oriented projects are perceived as most likely to be available by the MINT students however (MINT 43.6%; HM 39.1%; Ø 36.4%).

A further indication of the practical relevance of a course is a high *level of contact with persons* working in the later professional field, which was most likely to be mentioned by HM (HM 23.9%; Ø 12.5% for “frequent” contact; no figure). MINT students, in particular, rarely or never have (70.8%) contact with persons working in their field (Ø: 64.4%; HM: 38.9%).

**Figure 5:**
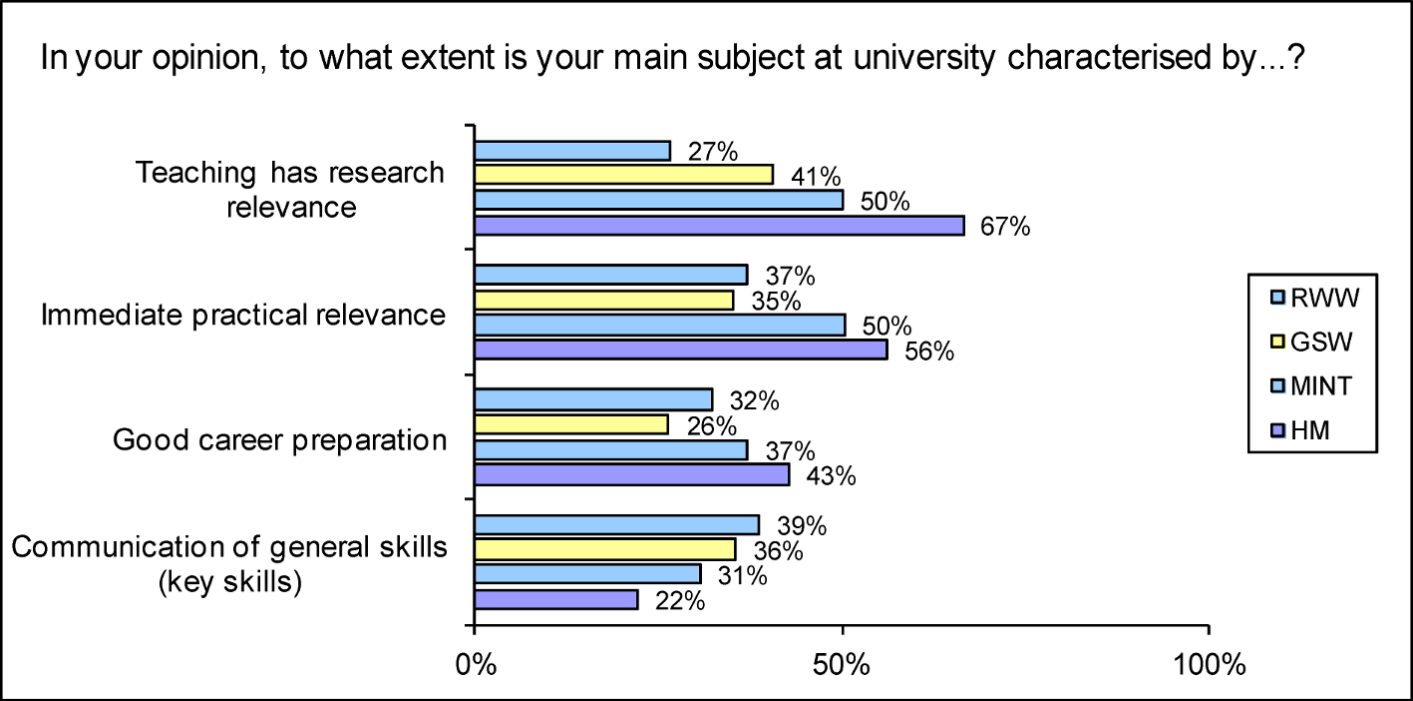
Selected responses to the question “In your opinion, to what extent is your main subject at university characterised by" (Proportion of scores 4-6 on a scale from 0-6 where 6=”very strongly”)

**Figure 6:**
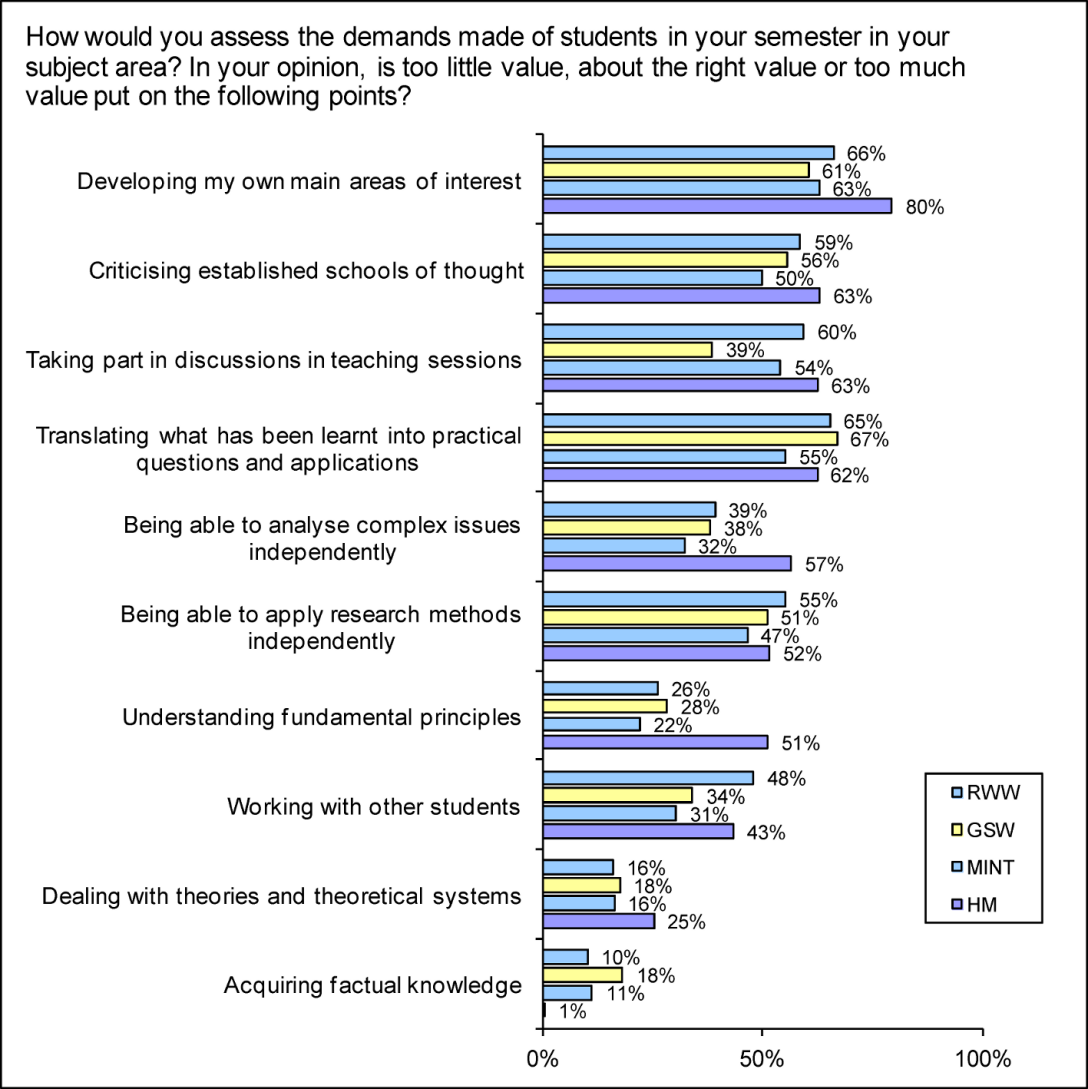
Responses to the question “How would you assess the demands made of students in your semester in your subject area? In your opinion, is too little value, about the right value or too much value put on the following points?" (Proportion of responses in the categories “much too little” and “too little”)

In a global assessment of their own course of study, students of human medicine in particular named high* practical relevance* (HM 56.1%; Ø 42.4%; see Figure 5 (Fig. 5)) and career preparation (HM 42.7%; Ø 32.5% indicate a tendency to ‘characteristic’). On the other hand, 62.4% of HM think that too little value is placed on translating *what has been learnt into practical questions and applications* (Ø 62.1%, see Figure 6 (Fig. 6)). The MINT disciplines performed best in this assessment.

**Figure 7:**
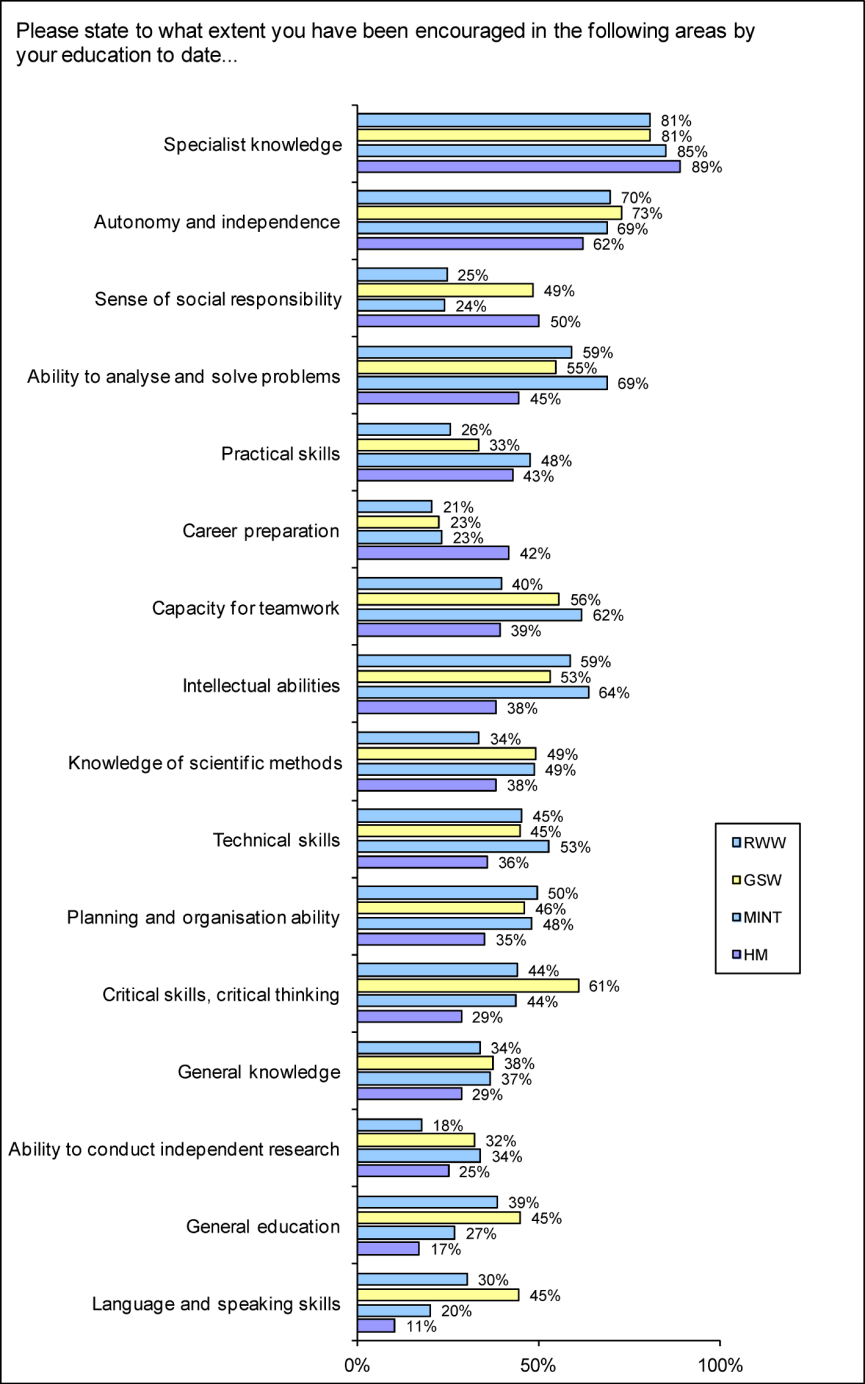
Responses to the invitation "Please state to what extent you have been encouraged in the following areas by your education to date." (Proportion of scores 4-6 on a scale from 0=not encouraged at all to 6=greatly encouraged)

**Figure 8:**
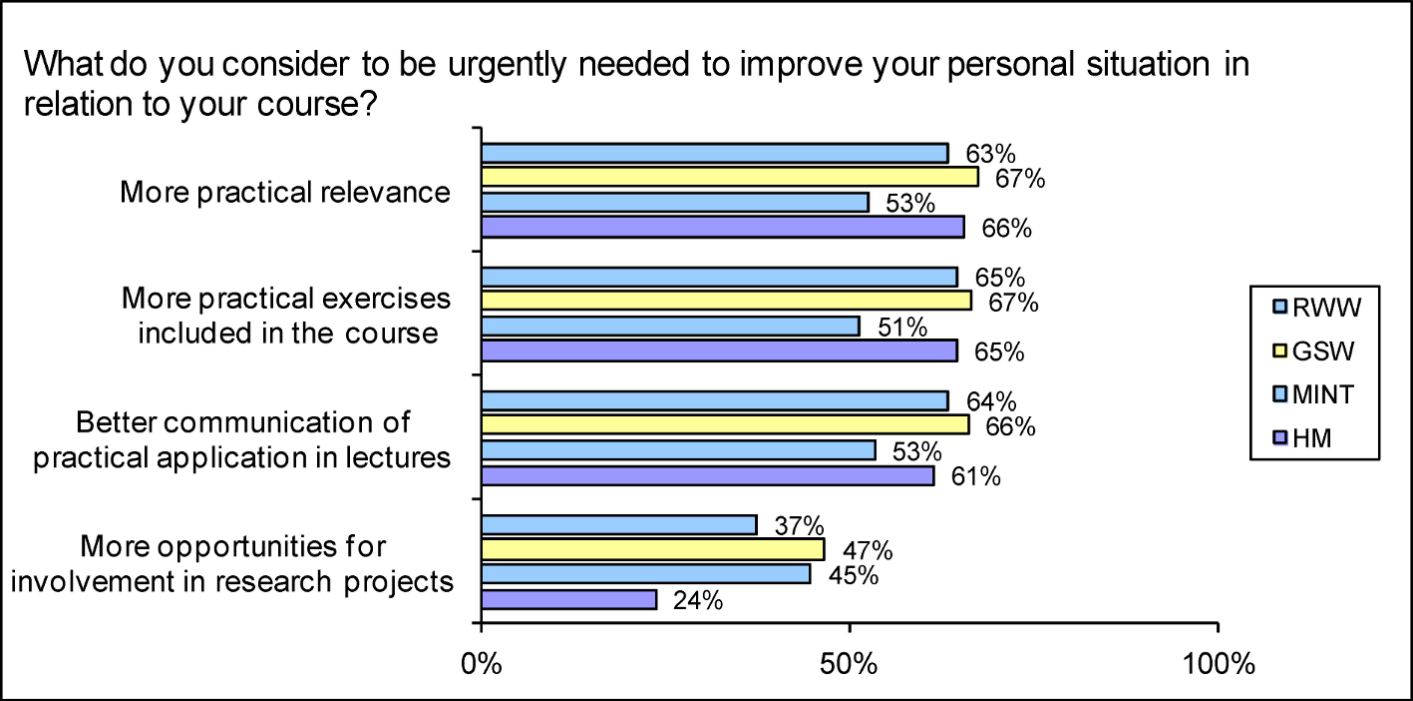
Responses to the question “What do you consider to be urgently needed to improve your personal situation in relation to your course?" (Proportion of scores 4-6 on a scale from 0-6 where 6=“most urgently needed”)

A higher than average proportion of MINT students (47.7%) and HM students (42.8%) feel that they are supported in developing* their practical skills* (Ø 37.7%; see Figure 7 (Fig. 7)). Humanities and social sciences students in particular would like more *practical relevance* (67.4%; see Figure 8 (Fig. 8)). Many human medicine students (65.6 %) also share this view (Ø 61.1%). *More practical exercises* as part of the course are required by the GSW students in particular (66.5%; Ø 60.6%), with students of RWW (64.7%) and HM (64.6%) a close second.

#### Promoting scientific skills

Students of human medicine in particular consider their education to have *research relevance* (HM 66.5% Ø 42.9%; see Figure 5 (Fig. 5)). This trend is repeated at a somewhat lower level in the assessment of whether *questions relating to current research* are incorporated into the sessions (HM 49.6; Ø 39.5% indicate a tendency to ‘frequently’). In response to the question whether courses are offered on *research methods *(HM 33.8%, Ø 27.7%), o*n current research* (HM 33.1%, Ø 32.1%) and on *practical introduction to research* (HM 28.8%; Ø 24.7%), it was HM students and MINT students who were most likely to agree that they were (see Figure 4 (Fig. 4)). However, only 1.2% of the medical students “very frequently” and 9.9% “frequently” attempt to find out how a *research result* was obtained (Ø 2.6% and 12.8% respectively). They are also less likely than average to be introduced to the *use of research* methods by those teaching them (HM 10.4%; Ø 14.8% for “most” and “all” teaching institutions) – this is most likely to occur in the case of the GSW (19.4%), and least likely for the RWW (7.4%). HM students, however, are the most likely to have assisted with a *research project at university* (HM 26.8%; MINT 15.4%; GSW 12.5%; RWW 6.0%; Ø 13.2%; no figure). They are the least likely to consider it of high importance to offer more *opportunities for involvement in research projects* (HM 23.7%; Ø 42.7%; see Figure 8 (Fig. 8)).

An indicator of successful scientific orientation is whether students are encouraged to undertake independent scientific work. MINT students are the most likely to conduct their *own experiments or studies*; 4.6% of them do so very frequently and 12.1% frequently (HM 2.7% and 8.8%). More than half of human medicine students (50.7%) state that they have never conducted experiments or studies (Ø 43.9%). Accordingly, 51.6% of the HM think that *being able to apply research methods independently* is assigned too little significance in their education (Ø 50.3%; see Figure 6 (Fig. 6)).

As a result, the ability to conduct *independent research* is encouraged, particularly from the point of view of the MINT students (34.1%; see Figure 7 (Fig. 7)), closely followed by the GSW (32.4%), while the HM are below average (25.1%; Ø 29.7%). More than half the students (HM 54.5%; Ø 53.3%) are more likely to feel that their ability to conduct research is not encouraged. Encouragement to acquire k*nowledge of scientific methods* is particularly stated by those questioned in humanities and social sciences (49.4%) and the MINT subjects (48.8%); in HM, students are less likely than average (38.3%) to feel encouraged to do this (Ø 45.3%). Accordingly, human medicine students were the least likely to state that teachers give *assistance/instructions in scientific projects* or in the writing of papers or similar activities (HM 17.2%; Ø 44.2%; GSW 56,6% indicate a tendency to ‘frequently’) or to encourage deep *involvement with scientific problems* (HM 16.0%; Ø 20.0%; GSW 24.9%; for “most” and “all” teaching institutions, no figure).

#### Promoting generally applicable skills

Practical and research skills should be constructed on theoretical foundations. MINT students are comparatively satisfied (50.3% “just right”) with their ability to deal with theories and theoretical systems. This satisfaction is shared by only 35.8% of HM students (Ø 47.0%). An above-average number of human medicine students think that too little value is placed on dealing with theory (HM 25.3%; Ø 17.3%; see Figure 6 (Fig. 6)) – but almost as many think that too much value is set on it (HM 23.3%; Ø 27.1%).

To the question of how far they have been *encouraged in individual subject areas to date*, those questioned most frequently respond positively with regard to their specialist knowledge (see Figure 7 (Fig. 7)). In HM, technical *skills and systematic working (HM 35.9%; Ø 47.2%) and planning and organising ability* are least frequently encouraged (HM 35.2%; Ø 46.8%).

Human medicine students are the least likely to feel encouraged in their* general education/breadth of knowledge *(HM 17.2%; Ø 35.5%), in *generally applicable knowledge/interdisciplinarity *(HM 28.7%; Ø 35.9%) and in their capacity for *teamwork and/or cooperation and ability to perform tasks with others *(HM 39.3%; Ø 53.8% indicate a tendency to being supported). Accordingly, HM students are the most likely to state that communication of *generally applicable skills* is not typical of their subject (49.6%; Ø 37.9%; see Figure 5 (Fig. 5)); prospective lawyers and economists (48.0%) and the human medicine students (43.4%) are more likely than average to voice the criticism that too little value is put on *cooperation with other students* (Ø 36.2%).

It is important for the capacity for teamwork and learning effects to be able to work discursively with other people. Almost two thirds of human medicine students (62.6%) find that too little value is placed on *discussions in teaching sessions* (Ø 49.9%; see Figure 6 (Fig. 6)), with the same applying to expressing *criticism of established schools of thought* (HM 63.1%; Ø 54.6%; indicate a tendency to ‘too little’). Human medicine students are the least likely to state that they feel encouraged in their *language and speaking abilities/ability to participate in discussions* (HM 10.5%; Ø 30.7%) and in their *critical ability/critical thinking* (HM 28.7%; Ø 49.3% encouraged). They are the least likely to receive feedback on *the results of tests, examinations or coursework* (HM 66.9%; Ø 53.4% indicate a tendency to ‘none’). 34% of human medicine students (Ø 16.8 %) even state that they had “never” received explanations.

Human medical students are the least likely to develop their own thoughts in *problem solving* (HM 4.3% “very frequently”, 25.9% “frequently”, 6.8% “never”; Ø 10.0%; 32.2%; 5.1%; no figure) and feel the least supported in the analysis of problems and problem-solving ability (HM 44.7%; Ø 60.0% encouraged; see Figure 7 (Fig. 7)). The human medicine students also feel the least likely to be encouraged in the following qualities:

- *autonomy and independence* (HM 62.1%; Ø 70.0% encouraged)
- *intellectual abilities*, i.e. logical methodical thinking (HM 38.3%; Ø 56.9% encouraged) or they are most likely to rate the support given as too little
- *independent analysis of issues* (HM 56%; Ø 37.5% indicate a tendency to too little; see Figure 6 (Fig. 6)) and
- *understanding fundamental principles *(HM 51.2%; Ø 27.2% indicate a tendency to too little).

In contrast, medical students in particular state that they have to learn too much factual knowledge (HM 74.1%; Ø 35.2% indicate a tendency to too much). 26.4% even choose the option of the extreme response “much too much” (Ø 8.3%; see Figure 7 (Fig. 6)).

## Discussion

This analysis is conducted on the basis of one written representative survey to find out what expectations students of human medicine have of their medical education and profession, the extent to which they feel encouraged to develop scientific and practical skills and to what extent they differ in this from students in other disciplines.

### Data base

The 11^th^ Student Survey, which involves more than 7500 participants, enables discriminating analysis. The response rate (about 28%) and the disproportionately high participation by women in the response are also found in similar surveys (see [22], [23], [24]). The 11^th^ Student Survey was conducted in 25 tertiary education establishments and does not therefore include the whole breadth of university courses offered in Germany. The responses from the human medicine students were obtained from 10 faculties, each of which puts different emphasis on research and practice as part of their model or conventional curriculum. The results always represent the subjective perspective of the students. Factors that are not explicitly included in the student survey may colour their assessments. Those questioned are still studying and have not reached a “final” conclusion on how well the practical and scientific orientation of their studies will continue to serve them. Their responses are influenced to some extent by what they think their professional life will actually be like. The picture should be completed by surveys of people who have completed their medical education.

2146 students (28.5% of all those surveyed) are studying more than one subject simultaneously so that the responses to some items could include assessments relating to other subjects. Professional and educational objectives may change during education. There are only minor differences in the period of study in the four disciplines, however, which means that a comparable picture emerges. The information given on the general benefit of tertiary education and on significant determinants of profession was not the subject of specific enquiry according to respondents’ own subject area. It can be assumed, however, that the responses reflect the background to the student’s choice of subject. With regard to the question analysed “What do you see as the benefits of higher education to you?” (see Figure 1 (Fig. 1)) it is not possible to conclude unequivocally whether the items selected (e.g. income, high social position) are considered worthwhile subjectively or whether they are seen as an incidental result of university study. When all questions analysed are combined, however, interdisciplinary differences are identified relating to the expectations and aims of university education and profession. It is, however, also reasonable to assume that within each of the disciplines examined here (even within a single course of study) heterogeneous expectations of the course, diverse understanding of theory and practice and different professional horizons of experience are present (see [24]).

#### Results

How important the aim of working with and for people is for human medicine students has already been indicated in earlier studies [16], [17]^2^. This analysis demonstrates that this feature is significantly more important for human medicine students compared to those studying other subjects. The former are by far the most likely to be prepared to take on duties involving a high degree of responsibility (see Figure 2 (Fig. 2)). In contrast to law and economics students in particular, they are substantially less likely to be motivated by external career characteristics such as management position or income; and in contrast to the humanities students, they are less likely to study the subject for its own sake (e.g. for general education, intellectual and personal development). Human medicine students in the subject comparison are more strongly motivated in their choice of subject by practical activity in their profession.

There are two ways to explain the fact that the human medicine students have lower than average expectations of becoming a person with a good general education because of their studies, yet have an above-average belief that the rapid conclusion of their studies would benefit them in their personal and intellectual development. This view could

1. Be based on their experience of their studies to date, in that rote learning of specialist knowledge is more dominant than is necessary in the subject comparison and/or
2. Be caused by their particularly frequent firm career aspirations, i.e. for them studying is predominantly a means to an end, entering a profession.

In addition they have one of the longest standard periods of study before them. Their clear career aspirations combined with their above-average interest in a variety of possible professions indicates that they value the wide choice of professional specialisations above all, because only in very rare cases do they consider working outside medicine [25], [26]. Overall, human medicine students are particularly likely in comparison with other subjects to expect their medical education to prepare them for practical professional activity.

Of all the groups, MINT students put the most weight on scientific focus (exploring the unknown, scientific work). Human medicine students may also assume that they are pursuing research in their curative activity – although they are not exactly discovering the “unknown”, as specified in the way the item was worded. That it is of less than average importance to the HM to implement their own ideas also points to this. The humanities and social science students take leading place in relation to this point. The average interest only in scientific work taken by the human medicine students contrasts with the above-average rate of doctorates [27], which probably tends to occur from extrinsic motives.

It is also evident in other studies that MINT students are more likely to be scientifically oriented and law and economics students more likely to be focussed on practice or explicitly on their profession than students in other subject groups [6], [17]. Qualitative surveys could help develop a deeper understanding of the meaning that students assign to the central concepts “knowledge” and “practice”.

Although human medicine students express especially high expectations that their medical education will prepare them for a profession, they are comparatively satisfied with the way their study is practice-oriented. These good scores in the subject comparison may be based on the fact that the study of medicine more than other courses of study prepares students for a concrete (even though varied) professional field and that (obligatory) internships have to be completed particularly frequently (as in [23]). Even when the students value the benefit of their internships highly [18], the acquisition of practical skills in the extracurricular four-month clinical traineeships is often unsystematic and non-reflective and associated with lack of feedback on performance (see [28], [29], [30]). A high level of contact on the part of HM with professionals in their field can be increased by physicians in their own families (on the profession’s self-recruitment see [31], [16]).

Even though the human medicine students are more likely than average to feel supported in developing their practical skills (43 % indicate a tendency to be supported), more than half, however, regret that what is learnt is not put into practice in practical issues and applications; one in three does not feel supported in developing practical skills and more than one in four does not have a sense of being prepared for a profession. There is also the fact that the students of human medicine are more likely than average to consider (even) greater practical relevance as an urgent consideration. While entry into professional life puts some expectations that practical skills will be communicated into perspective [32], surveys of practising physicians [33], [34], [35] also reveal deficits in practical skills. The status of organising ability identified in the Student Survey as in need of improvement makes entry into professional life more difficult (see Figure 7 (Fig. 7) and [33]). Low scores from the prospective physicians for the promotion of *language and speaking abilities* are unfavourable, because communication is a substantial part of their work, with impact on patient satisfaction and quality of care [36], [37], [38]. The low uptake of interdisciplinary programmes by medical students, the below-average support of their general education, the paucity of contacts with students from other disciplines and the fact that regular sessions are less likely than average to be organised on an interdisciplinary basis in medical education from the students’ point of view and encourage less than average capacity for teamwork, do not correspond to a professional reality that is marked by *interdisciplinarity and team work* (see [39], [40]). After completing their studies, human medicine students perceive themselves in the subject comparison as possessing below-average social skills [34]. It is vital, therefore, to incorporate suggestions (see [41]) and tried and tested examples into medical education, with the aim of promoting social skills, interdisciplinarity and capacity for team work [42], [43], [44], [45].

The proportion of students that feels encouraged to develop practical skills is highest among the MINT students. Because reports of internships and contact with people in their later professional field are comparatively rare, a specific advantage of their higher education appears to be that they can enhance their skills, in particular through their own practical experimentation as part of *regular teaching sessions*.

Human medicine students are the most likely to consider their medical education to be *research-oriented* and are the most likely to be involved in research projects. The extent to which this point of view is occasioned by their working on their own doctorate, which often accompanies their medical education, cannot be identified from the data. When 45% participate in research projects, but only 29% are given a practical introduction to research and only 25% feel encouraged to develop their research skills, some degree of incongruity becomes evident. En détail, the MINT students are the most likely of all groups to describe that they are encouraged to develop their research skills and to undertake independent research; the humanities students, too, are given better support in developing academic skills. The results indicate that scientific work in the education of medical practitioners is stronger on the passive theoretical level (e.g. by reference to research results in lectures), but less likely to be actually attempted.

Retrospectively as well, in a graduate survey, human medicine students were more likely than average to evaluate the item “Practice in academic working methods” in their studies [34] as good and their knowledge and skills in academic methods as substantially poorer than average among those surveyed. A below-average 38% of medical graduates have, in their opinion, the ability to put scientific concepts/results into practice ([34], S. 80). In addition, in this survey only 27% of the human medicine graduates state that they need knowledge of scientific methods in their later career. Many human medicine students do not appear to be aware that, even though their professional focus is not generally active research (see [25]), they use – or are supposed to use - scientific methods in their healing activity. On the other hand, human medicine graduates consider their less than average scientific competence in professional life, which also became clear in the student survey, to be problematic (e.g. deficits with regard to “Applying knowledge to new problems”, “Problem-solving ability”, “Interdisciplinary thinking”; [33]). It is equally alarming in the face of the marketing strategies used by suppliers of medical devices that the next generation of medical professionals only infrequently ask, according to the results of the Student Survey, how a research result is obtained. The German Council of Science and Humanities has accordingly required independent scientific work from every student of human medicine [46].

There are two explanations, which are to some extent complementary, as to why human medicine students are the least likely to feel supported in developing their intellectual abilities:

- The study of human medicine promotes intellectual abilities as much as other courses but this encouragement is perceived as less because of the degree of cognitive abilities and knowledge present before the start of medical education (the above-average marks in the German school leaving examination and self-evaluation of knowledge are indices for this according to Willich et al. [17]).
- Medical education is defined particularly strongly by the passive-receptive acquisition of knowledge and less by independent analysis, problem solving, discussion and scrutiny – this fits with the results of the previous sections.

Repeated student surveys show two positive trends. Among prospective physicians, the view that the *requirement to put what has been learnt into practice* is dosed about right has consistently gained agreement since 2001 [18]. They feel increasingly supported in their *autonomy and independence* as well. As these results show, their statements on the application of what they have learnt remain close to the average for all subjects and scores for autonomy are significantly lower. According to another study as well, the ability “to apply current knowledge to new questions and problems”, “critical thinking” and “autonomy and independence” are given below-average encouragement in the subject group “Medicine”^3^ ([47], p. 11f). This is unsatisfactory because of the responsibility that generally has to be assumed as early as the start of specialist training and is also inconsistent with professional motivation, because human medicine students show an above-average interest in making independent professional decisions (HM 91%; Ø 87%) and in carrying out duties involving a high degree of responsibility in their later career (HM 85%; Ø 69%) (see Figure 2 (Fig. 2)).

If the *critical faculties* of human medicine students are particularly rarely encouraged and they receive very little feedback on their own performance, the ability to learn from their own mistakes, which is essential, is made more difficult. Promoting the *discussion skills and critical faculties *of students and teachers – in accepting and giving criticism – can contribute to improving the quality of education, medical care and research. Subjective perception of the acquisition of competence is the higher, *“the more the teachers address themselves to the students”* or *“refer in their teaching to research and encourage students to do their own research*“ ([48], S. 174) or when the students perceive their study as possessing both a high practice orientation and pronounced scientific orientation [7], see also [6].

The students’ assessments can be caused by other factors that are not associated with the way their studies are organised. The restrictions on registration may mean that the human medicine group of students is more highly motivated but also more demanding than others. Particularly clear expectations of their later professional activity (including obligatory internships and above-average frequency of contact with potential employers) could render them particularly aware of their deficiencies in competence in the subject comparison. Independently of possible reasons for the differences between subject groups, it is clear that human medicine students would like more support particularly in developing social skills and independence. The good scores for global assessments of practice and research relevance in human medicine appear to be based predominantly on the fact that the students are the passive recipients of knowledge of practice and theory. Analysis suggests that, in the study of human medicine, independent thinking and active experimentation on the part of the students under experienced leadership should be more frequently integrated to enhance practical and scientific competence.

## Conclusion

The need for students of human medicine to have a practice-oriented course of study that prepares them for their profession should be particularly high because of their clear career objectives, their interest in practical work with and for people and their slightly less than average interest in more general education. Although they show slightly an above-average interest in scientific and average interest in research activity, the relevant steps to achieving this, such as putting their own ideas into practice, are less interesting to them than to the average student in the survey. The prospective physicians are probably not (yet) aware how important and interesting it is - as part of patient-related activity as well - to apply a scientific approach. In the context of the efforts of medical associations to standardise treatment in guidelines, this finding presents a challenge for transforming the next generation of physicians into professionals.

In the study of human medicine, practical and research relevance are comparatively well developed according to the students’ global assessment; in detail, however, various imperfections can be identified. For example, human medicine is awarded the worst scores of the four areas of study in promoting independent thinking, systematic working and social skills. It is not feasible to fill these gaps by additional teaching sessions. Performance requirements and workloads in human medicine are already rated as too high by the students [15]. What is needed, therefore, is to review traditional teaching content and to realign teaching and learning methods. The step that immediately suggests itself in relation to developing scientific skills is to progress from participation in research projects to dealing independently with scientific projects. Because physicians are frequently given a high level of responsibility very soon after completing their studies and the state of scientific knowledge advances rapidly, the independence promoted to date while studying is insufficient, both in practice and in theory. An exchange of experience with the MINT and GSW disciplines could provide the necessary stimulus.

## Notes

^1^ Our thanks to the Tertiary Education Research working group at the University of Constance for allowing us to use the Public Use File relating to the 11th Student Survey in the “Conditions of study and student orientation” project and to Dr. Michael Ramm for his helpful advice. The project was and continues to be sponsored by the German Federal Ministry of Education and Research (BMBF).

^2^ Willich et al. [17] analyse “medicine” only in accordance with official statistics i.e. including other health-related areas of study

^3^ This includes veterinary medicine etc. according to the Federal Statistical Office classification

^4^ The analysis differs from the data in Ramm et al. [19], because the data were cleaned differently and in addition the data from the Federal Statistical Office for the winter semester 2009/2010 were used for German and non-German students, whereas Ramm et al. refer to data from winter semester 2008/2009 and exclusively to German students.

## Competing interests

The authors declare that they have no competing interests.

## Supplementary Material

Longer version of Figure 2: Responses to the question “What is particularly important to you personally in a career?" (Proportion of scores 4-6 on a scale from 0= “completely unimportant” to 6= “very important”)

## Figures and Tables

**Table 1:**
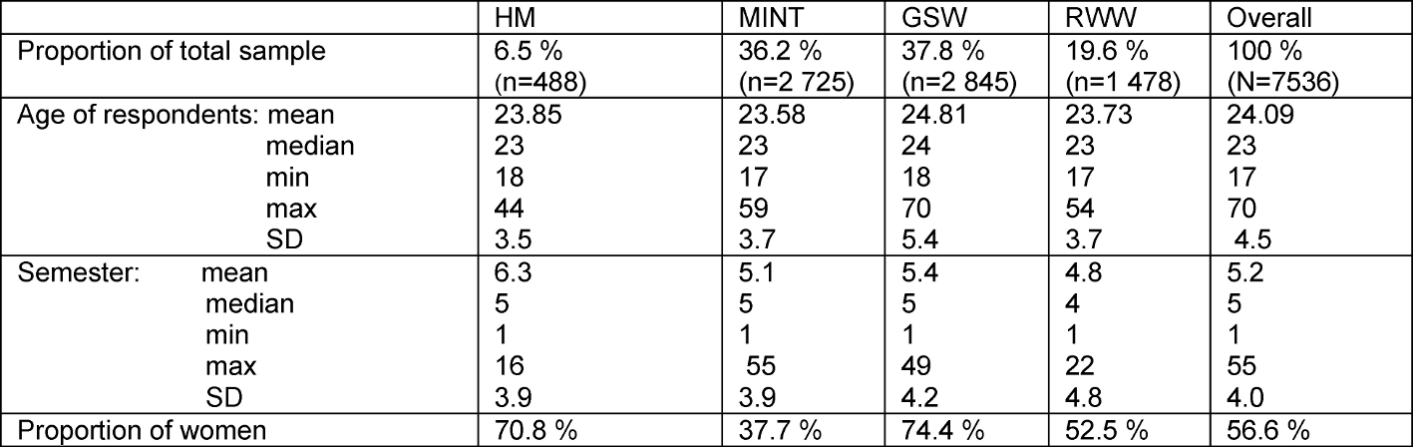
Sociodemographic characteristics of the sample in the student survey

**Table 2:**
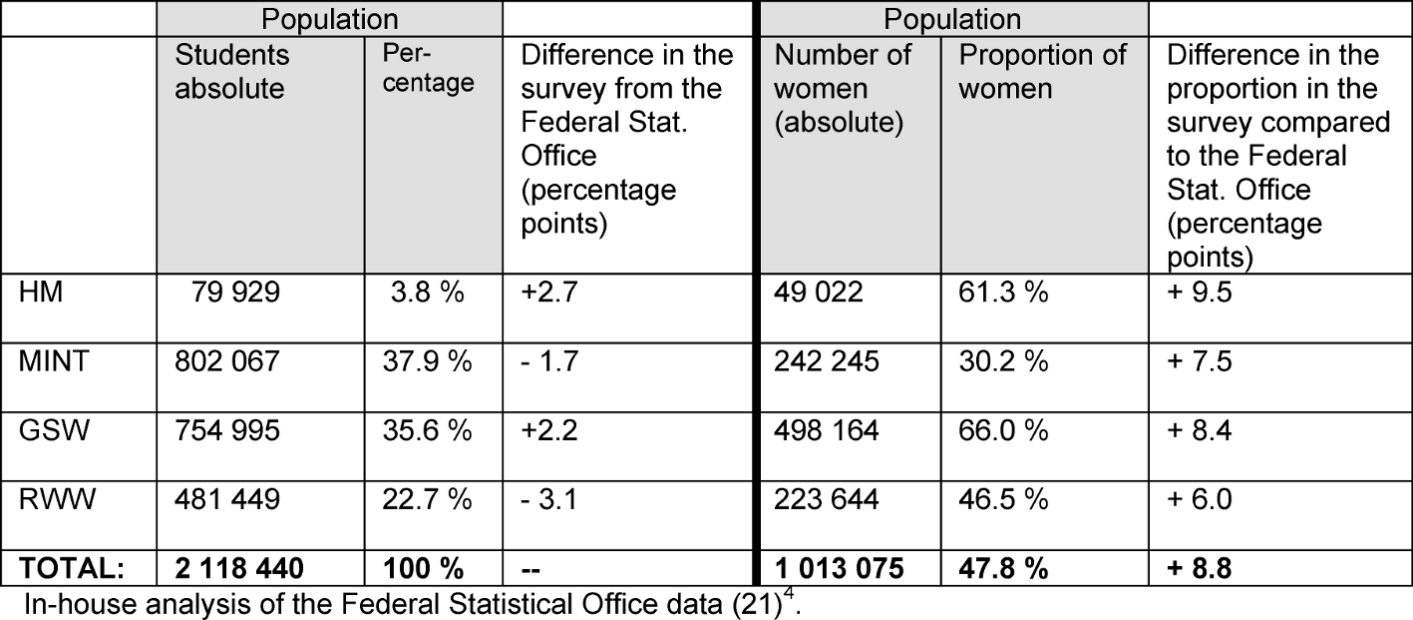
Comparison of the student survey with the population used by the Federal Statistical Office in 2009/10 by subject groups
