# Supplementary figures and images for: Theory and practice in medical education – expectations and development of skills experienced by students of human medicine compared with students in other disciplines
Source: GMS Z Med Ausbild. 2015 Feb 11;32(1):Doc8. doi: 10.3205/zma000950 (PMC4330638; doi:10.3205/zma000950)

# What is particularly important to you personally in a career?

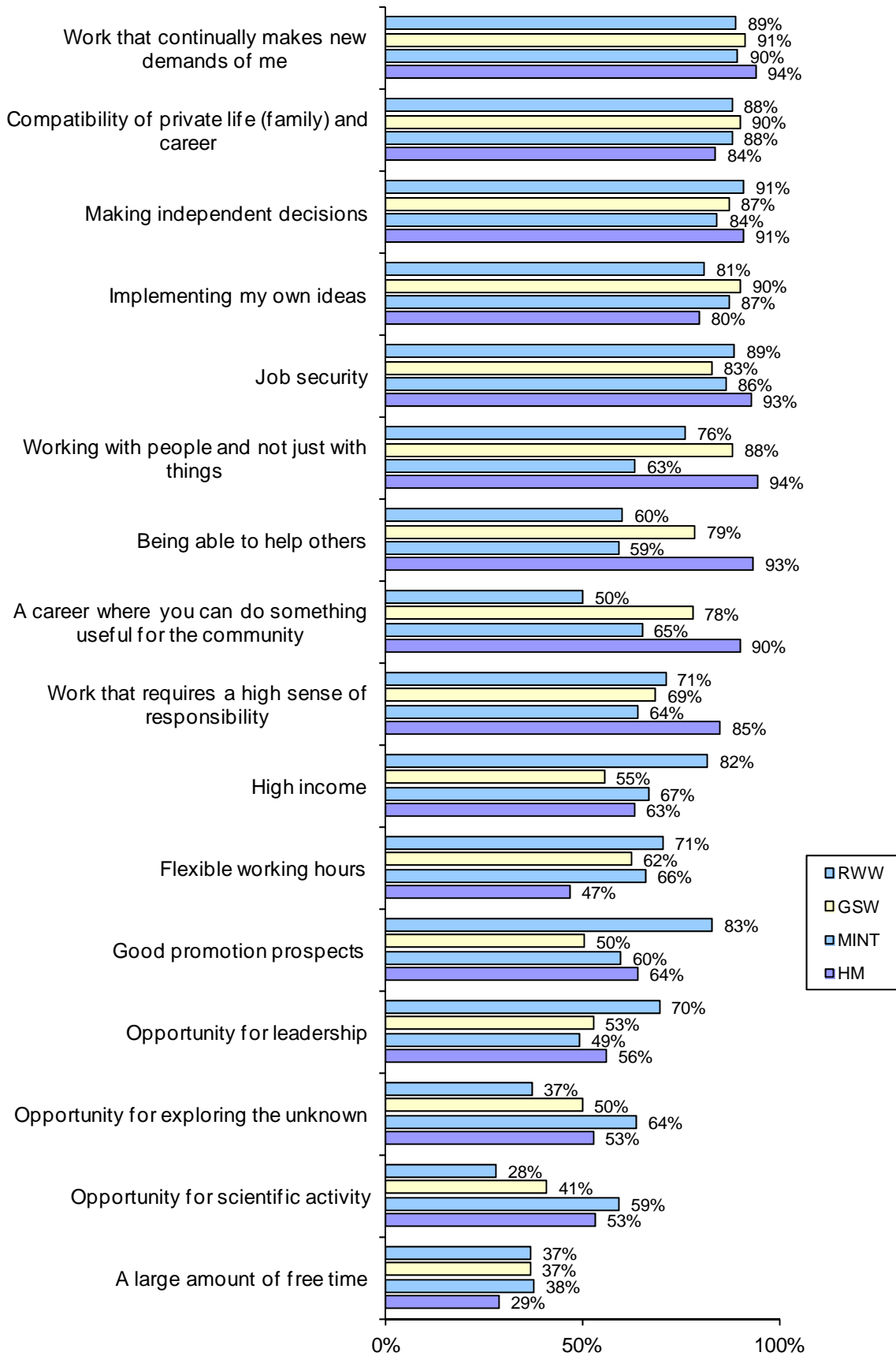

Supplement: Longer version of Figure 2: Responses to the question “What is particularly important to you personally in a career?" (Proportion of scores 4-6 on a scale from 0= “completely unimportant” to 6= “very important”) [file ZMA-32-8-s-001.pdf]
